# Extratemporal Malignant Nerve Sheath Tumor of Facial Nerve with Coexistent Intratemporal Neurofibroma Mimicking Malignant Intratemporal Extension

**DOI:** 10.1155/2015/790941

**Published:** 2015-08-09

**Authors:** Mitsuhiko Nakahira, Naoko Saito, Masashi Sugasawa

**Affiliations:** ^1^Department of Head and Neck Surgery, Saitama Medical University International Medical Center, 1397-1 Yamane, Hidaka, Saitama 350-1298, Japan; ^2^Department of Radiology, Saitama Medical University International Medical Center, 1397-1 Yamane, Hidaka, Saitama 350-1298, Japan

## Abstract

We present an extremely unusual case of an extratemporal facial nerve malignant peripheral nerve sheath tumor (MPNST) arising from preexistent intratemporal neurofibroma, illustrating a difficulty in discriminating between perineural spread of the MPNST and the preexistent intratemporal neurofibroma on preoperative radiographic images. The most interesting point was that preoperative CT scan and MR images led to misinterpretation that MPNST extended proximally along the facial nerve canal. It is important to recognize that the intratemporal perineural spread of neurofibromas and MPNST share common imaging characteristics. This is the first report (to our knowledge) of these 2 lesions coexisting in the facial nerve, leading to misinterpretation on preoperative images.

## 1. Introduction

The infiltration and extension of tumor along nerves have long been considered to be associated with malignancy, to be an important mode of tumor spread and a sign of increased aggressiveness [1–3]. The facial nerve may provide a route of spread along which extratemporal malignancies extend into the skull base and central nervous system. Preoperative images play a crucial role in early diagnosis and determining the extent of tumor extension [1–3]. In this report, we present an unusual case of extratemporal malignant peripheral nerve sheath tumor (MPNST) mimicking tumor extension proximally along the facial nerve canal on preoperative radiographic images.

## 2. Case Report

**Figure 1:**
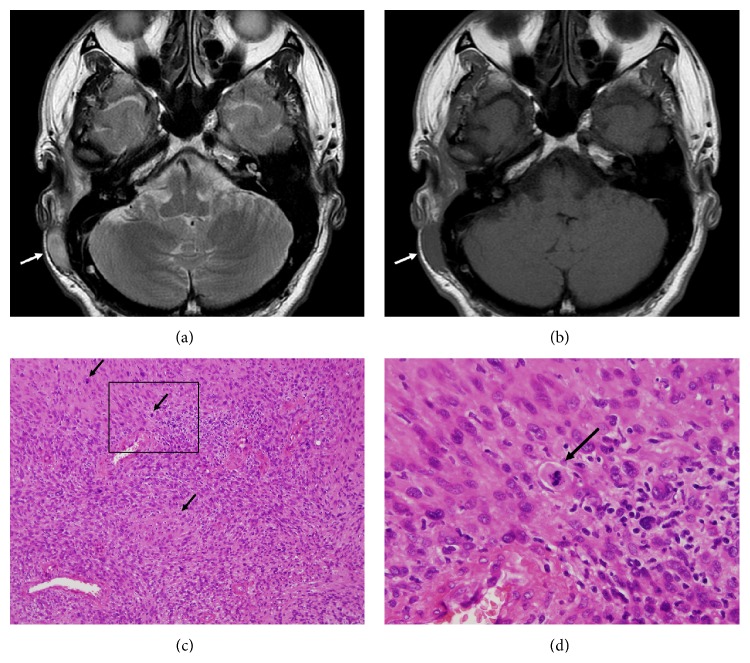
MPNST of the right postauricular region. (a, b) Axial T1- and T2-weighted MRI undertaken at the previous hospital shows a right postauricular elliptical tumor (arrow). (c) Photomicrograph of the resected tumor which is composed of spindle cells disposed in sweeping fascicles. The mitotic figures present are characteristic of malignancy (arrows) (Haematoxylin and Eosin stain; original magnification ×200). (d) Photomicrograph at higher power showing mitosis (arrow) (Haematoxylin and Eosin stain; original magnification ×600).

**Figure 2:**
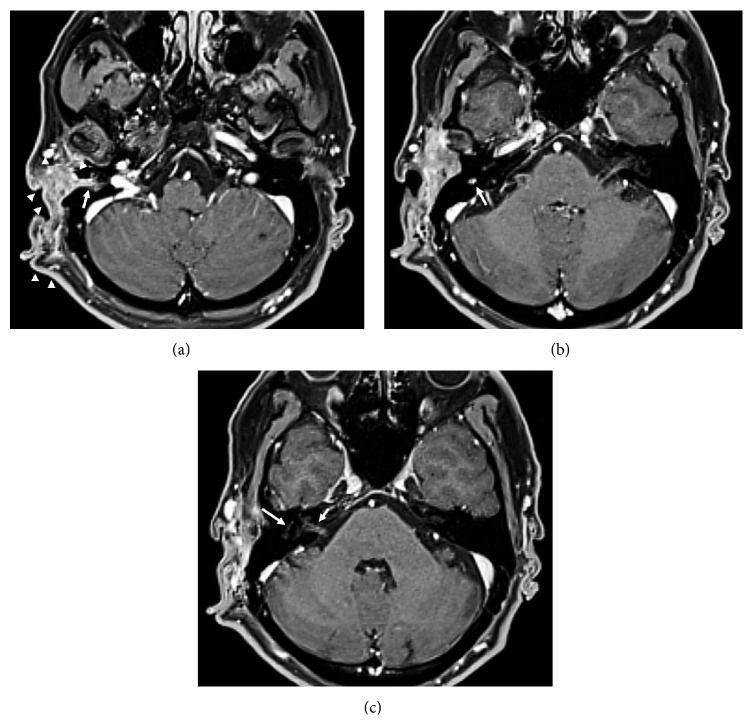
Axial contrast-enhanced T1-weighted MRI after partial resection of the postauricular MPNST. (a) Residual periauricular subcutaneous ill-defined lesions (arrow heads) extending radially from the stylomastoid foramen (arrow). (b) Enhancement of the mastoid segment of the facial nerve (arrow). (c) Enhancement of the tympanic segment (long arrow) and intracanalicular segment (short arrow) of the facial nerve.

**Figure 3:**
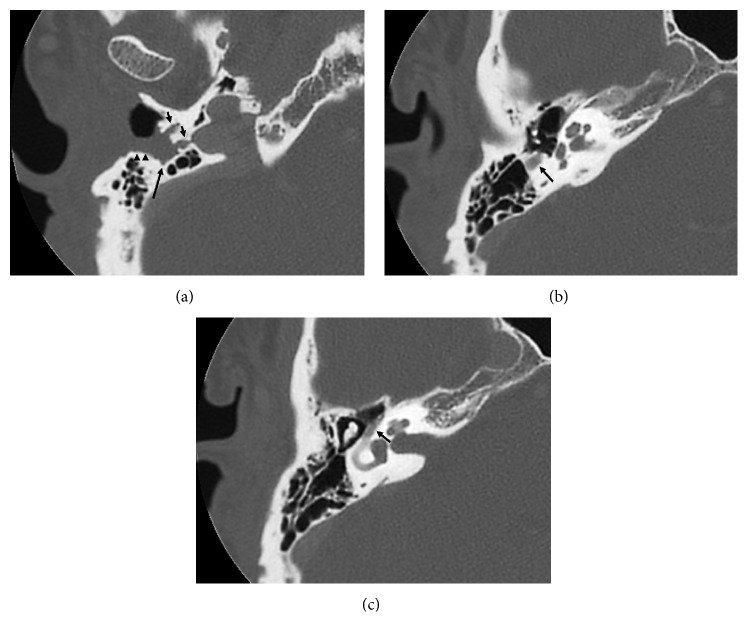
Axial CT images of the right temporal bone. (a) A mass in the stylomastoid foramen (large arrow) is destroying the tympanic bone and mainly spreading outside the bone (arrow heads). Note that, additionally, there is bony destruction on the inward side that might have spread along small branches of the facial nerve (small arrows). (b) The bony canal of the mastoid segment of the facial nerve is enlarged (arrow). (c) The bony canal of tympanic segment of the facial nerve is enlarged (arrow) as compared with the contralateral normal side (not shown).

**Figure 4:**
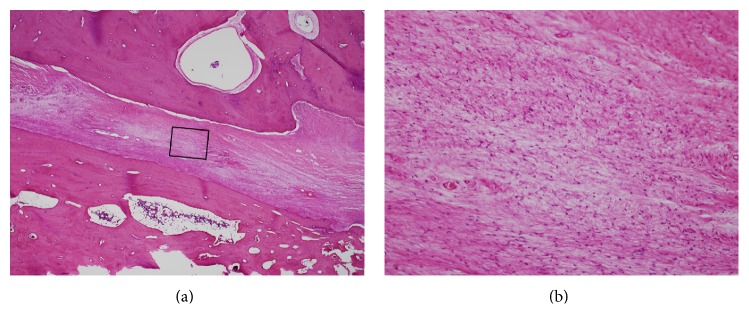
Histopathological findings of resected specimens. (a) Neurofibroma of the facial nerve in the mastoid segment of the osseous canal (Haematoxylin and Eosin stain; original magnification ×20). (b) Photomicrograph at higher power showing that the tumor is composed of cells with thin, elongated nuclei and scant cytoplasm, embedded in a myxoid stroma. Note that there is no evidence of malignancy (Haematoxylin and Eosin stain; original magnification ×200).

A 43-year-old male with type 1 neurofibromatosis (NF 1) was referred to our hospital because of an increasing subcutaneous tumor of the right postauricular region, which was partially resected with positive margin and histologically proven as MPNST at the previous hospital (Figures 1(a)–1(d)). The patient did not complain of right facial nerve paralysis or hearing impairment. Magnetic resonance imaging (MRI) demonstrated residual periauricular subcutaneous ill-defined lesions extending radially from the stylomastoid foramen and subsequent abnormal enhanced lesions in the intratemporal facial nerve from the stylomastoid foramen to the internal auditory canal (Figures 2(a)–2(c)). Computed tomography (CT) of the temporal bone showed the subcutaneous lesions from the enlarged stylomastoid foramen and the enlarged facial canal (Figures 3(a)–3(c)). On the basis of these findings, the patient was strongly suspected of having a MPNST that had spread proximally along the facial nerve to the internal auditory canal. In order to obtain clear surgical margins, CyberKnife stereotactic radiosurgery was undertaken with a single dose of 23 Gy to the right internal auditory canal. Thereafter, the patient was treated with subtotal temporal bone resection, right neck dissection, and reconstruction with the rectus abdominal muscle free flap. Postoperative histopathological examination demonstrated no residual MPNST without lymph node metastasis but neurofibroma involving the subcutaneous tissue and the entire portion of the facial nerve canal (Figures 4(a) and 4(b)). The patient developed dysphagia because of incomplete lower cranial nerve palsy, in addition to 7th and 8th cranial nerves loss. After rehabilitation and surgical treatment with laryngeal elevation and cricopharyngeal myotomy, he gradually resumed eating. Although postoperative adjuvant therapy was not delivered, there was no recurrence at follow-up 24 months after surgery.

## 3. Discussion

MPNSTs are rare, aggressive malignant spindle cell tumors with a poor prognosis. The overall 5-year survival rate for patients with MPNSTs ranges from 34% to 52% [4–6]. Most MPNSTs arise from neurofibromas or de novo on normal peripheral nerves. Malignant transformation of neurofibromas occurs in 2% to 5% of patients with NF 1 as compared with an incidence of 0.001% in the general population [4–6]. In the present report, the MPNST arose from a preexistent benign neurofibroma in a common fashion that is associated with NF 1. The head and neck are involved in 8% to 20% of cases, and the neck is the most common primary site in this region [4–6]. MPNSTs involving the facial nerve or its branches are even more exceptional [7, 8]. In 2003, McGuirt Sr. et al. [8] reported the first case of malignant transformation of an intraparotid facial nerve neurofibroma without intratemporal facial nerve involvement in a 43-year-old woman with NF 1. In contrast to extratemporal origins, neurofibromas arising primarily in the intratemporal facial nerve are extremely uncommon, with the first detailed report [9] of a patient of neurofibroma in the mastoid segment of the facial canal in 2002. Searching the PubMed database for articles published up to December 2014 using the following relevant two keywords: neurofibroma and “facial nerve,” we found additional nineteen cases [10–13] of the intratemporal neurofibroma since the first case was reported in 2002 [9]. As a result, we did not find any patient of the intratemporal neurofibroma with malignant transformation in the retrieved articles. Accordingly, this is the first report of a MPNST and a primary intratemporal preexistent neurofibroma coexisting in the facial nerve, leading to misleading imaging findings. Moreover, the neurofibroma in the present report that was observed spreading extensively in the facial nerve was more exceptional. Nevertheless, strangely, we did not identify any clinical neurological signs and symptoms related to the right facial nerve at presentation. The absence of the facial nerve palsy despite the existence of the facial canal abnormality seemed to be a key factor to differentiate between remaining benign and malignant transformation in this rare entity.

The mainstay treatment for a MPNST is radical resection. However, inaccessibility resulting from perineural spread may result in the poor prognosis [7, 14]. The facial nerve may provide a route of spread along which extratemporal malignancies extend not only into the temporal bone but also into the skull base and central nervous system. Ibrahim et al. [3] reported that perineural spread may be the only evidence of malignancy. It is important for the interpreting clinician to detect perineural spread before the initiation of therapy because its presence can result in alteration of extent of resection. Besides, extent of perineural spread can affect the decision of resectability of the tumor. Radiographic features of perineural spread include neural thickening and enhancement and foraminal widening [1]. All these were identified in our patient. Moreover, abnormal findings were seen along the facial nerve between the stylomastoid foramen and the internal auditory canal in the present report. Therefore, we required aggressive treatment with curative intent, because the pattern of spread in this patient implied a poor prognosis.

In clinical setting, it is important to discriminate MPNSTs from neurofibromas, because a neurofibroma usually preceded the onset of a MPNST particularly in patients with NF 1. A biopsy remains the gold standard for accurately differentiating between them. A biopsy of tumors in soft tissues such as an extratemporal region can be performed relatively easily. In contrast, it is difficult to perform a biopsy in a case of intraosseous tumors as seen in our patient, because it requires a considerable destruction of bone and carries an inevitable risk of dissemination of malignant tumor cells. Therefore, MRI features such as larger tumor size, peripheral enhancement, perilesional edema, and intratumoral cystic change were reported to be useful in distinguishing MPNSTs from neurofibromas [15]. However, while these imaging features are helpful for large tumors in extremities and trunk, they might be unable to function for small intraosseous tumors as seen in our patient. Remarkably, Klingebiel et al. [9] reported that a neurofibroma arising in the mastoid segment of the facial nerve showed perineural tumor growth via small branches of the facial nerve as suggested in our patient. In contrast to common belief that perineural spread may be the only evidence of malignancy [3], they provided definitive evidence for the perineural spread of benign neurofibromas in the facial canal. MPNSTs are likely to develop from neurofibromas in particularly patients with NF 1. We should recognize that it can be clinically difficult to differentiate between perineural spread of MPNSTs and preexistent neurofibromas in the facial nerve canal on preoperative images, because MPNSTs and neurofibromas share a common growth pattern of perineural spread.
